# 

**Published:** 1910-07

**Authors:** 


### BOOKS RECEIVED.

Manual of Midwifery. By Henry Jellett, B.A., M.D. (Dub. Univ.), F.R.C.P.I., L.M., King's Professor of Midwifery in the School of Physic, Trinity College, Dublin. Second edition. Octavo, pp. 1222. Illustrated. New York: William Wood & Co. 1910. (Cloth, $6.00.)

Index of Symptoms with Diagnostic Methods. By Ralph Winnington Leftwich, M.D., Late Assistant Physician to the East London Children's Hospital. Fourth edition. 12mo, pp. 451. New York: William Wood & Co. 1910. (Cloth, $2.25.)

The Conquest of Disease through Animal Experimentation. By James Peter Warbasse, Surgeon to the German Hospital, Brooklyn. 12 mo, pp. 190. New York and London. D. Appleton & Co. 1910. (Cloth, $1.00.)

Mortality Statistics for 1908. Ninth annual report. E. Dana Durand, Director Bureau of the Census.

Johns Hopkins Hospital Reports. Vol. 15. Baltimore: The Johns Hopkins Press. 1910

A Manual of Personal Hygiene: Proper Living upon a Physiologic Basis. By eminent specialists. Edited by Walter L. Pyle, M.D., Assistant Surgeon to the Wills Eye Hospital, Philadelphia. Fourth revised edition. 12mo, of 472 pages, illustrated. Philadelphia and London: W. B. Saunders Company. 1910. (Cloth, $1.50 net.)

Disorders of Metabolism and Nutrition. By Dr. Carl von Noorden, Professor of the First Medical Clinic, Vienna. Parts VIII and IX. Inanition and Fattening Cures; Technic of Reduction Cures and Gout. 12 mos, pp. 103 and 112. New York: E. B. Treat & Co. 1910. (Cloth, $1.50 each.)

Transactions of the fifteenth annual meeting of the American Laryngological, Rhinological, and Otological Society, held at Atlantic City, N. J., June 3-5, 1909. Thomas J. Harris, M.D., Secretary, New York.

Borderland Surgery. By Gustavus M. Blech, M.D., Professor of Clinical Surgery, Medical Department Loyola University, Chicago. 12 mo, pp. 219. Philadelphia: Professional Publishing Co. 1910. (Cloth, $1.50.)

The Evolution of Antiseptic Surgery. Formulary of fine products issued by Burroughs Wellcome & Co., London (Eng.). 35-39 West 23d St., New York, N. Y.

Progressive Medicine, Vol. 12, June, 1910. A Quarterly Digest of Advances, Discoveries and Improvements in the Medical and Surgical Sciences. Edited by Hobart Amory Hare, M.D., Professor of Therapeutics and Materia Medica in the Jefferson Medical College of Philadelphia. Lea & Febiger, Philadelphia and New York. (Per annum, paper bound, $6.00; cloth, $9.00.)

### ITEMS.

### INDEX FOR VOL. LXV.

### AUGUST, 1909—JULY, 1910

Page

ADDRESS to graduating class, U. of B. .................... 639
Anesthesia, conscious .......... 510
venous ............. 552
Angina pectoris ................ 311
Antispitting ordinance ........... 676
Appendicitis ..................... 419
Arhovin in gonorrhea........... 259
Arteriosclerosis ................. 416

BEAHAN, A. L., appendicitis .. 419
Benzoate of soda, report on 117
Billings, William H., relation of local skin lesions to flat foot.. 66

**Books and Authors:**

Abbott: Bacteriology ....... 277
Abrams: Spondylotherapy ... 635
Abrams. Diagnostic therapeutics ...................... 680
Abrams: Diagnostic therapeu-
Aikens: Studies for nurses.. 404
A.M.A.: Proprietary. Medicines ..................... 520
A.M.A.: Handbook of therapy 520
Anders: Practice of medicine 519
Arnold: Diet charts ........ 281
Arny: Pharmacy ............ 220
Ashton: Gynecology ........ 462
Bacon: Otology ............ 337
Bailey: Embryology ........ 52
Ballenger: Diseases of nose, throat and ear ........... 344
Bandler: Medical gynecology 287
Bartley: Chemistry ......... 634
Bergey: Hygiene ........... 280
Bishop: Heart disease ...... 633
Blakiston: Visiting list, 1910. 283
Brewer: Surgery ........... 334
Bruck: Diseases of nose, etc. 682
Bryant: Surgery, vol. 6 ...... 221
Butler: Internal medicine ... 336
Cabot: Physical diagnosis... 283
Calkins: Protozoology ...... 278
Chapin: Diseases of children. 342
Clarke: Vital economy ...... 632
Craig: Malarial fevers ...... 227
DaCosta: Modern surgery .. 635
Deaderick: Malaria ......... 402
Dieffenbach: Hydrotherapy .. 115
Dorland: Dictionary ....... 115
Dorland: Dictionary ........ 225

Page

Edwards: Practice of medicine ....................... 276
Eisendrath: Surgical diagnosis ..................... 342
Esenwein: Writing short story ..................... 55
Evans: Obstetrics ........... 227
Fox: Treatise on ophthalmology ...................... 633
Friedenwald: Dietetics for nurses .................... 114
Friedenwald: Diet in health and disease ............... 169
Garceau: Tumors of kidney.. 339
Gould: Visual function, vol. 6 275
Green: Cancer ............. 222
Greene: Medical diagnosis... 576
Hall: Nutrition ............. 635
Hare: Typhoid fever ........ 343
Hare: Progressive medicine, June, '09, 52; Sept., 222; Dec., 403; March, '10 ..... 576
Hare: Practical therapeutics. 279
Haubold: Operative treatment ..................... 681
Head: Practical medicine series, vol. 1, '09, 54; vol. 2, 55; vol. 3, 171; vol. 4, 223; vol. 5, 226; vol. 6, 277; vol. 7, 337; vol. 8, 336; vol. 9, 461; vol. 10 .............. 577
Hill: Histology ............. 405
Hirschman: Diseases of rectum ...................... 169
Hirst: Textbook of obstetrics 340
Hoffman: Insanity law, N. Y. 404
Hort: Tuberculosis ......... 226
Howell: Physiology ........ 461
Hyde: Diseases of skin ..... 338
Jackson: Ophthalmic yearbook, vol. 6 .............. 226
Judd: x-ray ................. 282
Keen: Surgery, vol. 5 ....... 462
Kelly: Myomata of the uterus 228
Kerley: Diseases of children. 170
Keyes: Diseases of genito-urinary organs ............ 679
Klebs: Tuberculosis ........ 284
Knopf: Tuberculosis ........ 228
Lea & Febiger: Visiting list, 1910 ...................... 341
Lewis: Anatomy for nurses.. 578
Lippincott: International Clinics, 19th series, vol. 2, 55; vol. 3, 278; vol. 4, 516;

Page
20th series, vol. 1 ........ 577
Lusk: Nutrition ............ 636
McFarland: Pathogenic bacteria ...................... 461
McKenzie: Exercise ........ 224
Marrs: Confessions of a neurasthenic .................. 683
Martin: Surgical diagnosis .. 285
May: Diseases of eye ....... 231
Meyer: Bier's hyperemic treatment .................. 225
Modern Clinical Medicine: Diseases of children ...... 403
Monell: Electric currents ... 518
Ogden: The urine .......... 404
Osler: Modern Medicine, vol. 6 ..................... 113
Parke-Davis: Therapeutics .. 171
Ritchie: Human physiology . 171
Ritchie: Sanitation ......... 460
Saleby: Parenthood ........ 283
Savage: Hysteria ........... 223
Saxe: Examination of urine.. '634
Schorer: Vaccine and serum therapy ................... 170
Senn: Principles of surgery . 682
Simon: Chemistry .......... 281
Sluss: Emergency surgery... 683
Starr: Nervous diseases ..... 334
Stevens: Materia medica .... 284
Stimson: Fractures ......... 682
Strauss: Gout .............. 225
Taylor: Account book ...... 340
Thompson: Dietetics ....... 285
Thornton: Dose book ....... 405
Treves: Operative surgery, vol. 1, .................. 53
Tuley: Diseases of children.. 341
Tyson: Practice of medicine. 336
Von Neusser: Respiration and circulation ............... 224
Warbasse: Medical sociology 278
Webster: Diagnostic methods.286
Wharton: Minor surgery .... 279
Wilson: Medical diagnosis.. 338
Witthaus and Becker: Medical jurisprudence, vol. 3... 230
Wolff: Medical chemistry ... 114
Wood: Visiting list, 1910 .... 282
Wood: Microscopical diagnosis ..................... 335

**Reports:**

Commissioner of Education, vol. 1, 1908, 56; vol. 2, 228; vols. 1 and 2, 1909 ........ 632
Henry Phipps Institute, 1907-08 ...................... 576
Surgeon-general public health and marine hospital service, 1908 ...................... 55

Page

**Transactions:**

American Dermatological Association, 1908 ............ 517
American Laryngological Association, 1909 ............ 517
American Otological Society, 1909 ...................... 519
American Surgical Association, vol. 27 .............. 518
American Urological Association, 1908 ................. 518
Section on Obstetrics, A.M.A., 1909 ...................... 517

Books received: 56, 115, 172, 231, 287, 344, 405, 463, 520, 578, 636, 683
Bowerman, E. A., Buffalo Academy of Medicine, 1908-09 ..... 59
Bowling alleys ................ 452
Brain tumor, two cases of ....... 5
Breathe, how to ............... 626
Brown, William M., toxins and the liver ..................... 590
Buffalo Academy of Medicine.... 59
Buffalo delegation to A. M. A. ... 675
Bull, W. Sheldon, solution of milk problem ..................... 233
Burns, x-ray .................. 386
Butler, Glentworth R., angina pectoris ..................... 311

CALOX ..................214, 676
Cancer of rectum, surgical treatment of .......... 127
uterine .................. 544
in fishes ................ 625
Cannaday, J. E., early diagnosis of gallstone disease ........... 253
Carnegie foundation report ..... 675
Cholecystitis, chronic ........... 10
Collargol in puerperal fever ..... 41
in typhoid fever ....... 40
treatment ..........102, 491
Chase, Walter B., treatment of inoperable uterine cancer ....... 544
China, medical man of .......... 477
Coal tar products ............... 440
Cold storage time limit .......... 571
Colds unnecessary .............. 510
Cystitis, treatment of ........... 100

**College and Hospital Notes:**

Adam, J. N. Memorial Hospital ..................... 574
American Hospital (Paris)... 274
Buffalo College of Pharmacy 216
Buffalo General Hospital, 168, 274, 334, 459, 515 .......... 575
Buffalo Hahnemann Hospital 402
Buffalo University ..274, 460, 631

Page

Children's Hospital .216, 515, 575
College City of New York... 168
District Nursing Association. 575
German Deaconess's Home .. 51
Harrington Hospital for Children ..................... 51
Harvard University ......... 51
Johns Hopkins University .. 51
Marine Hospital, Buffalo .... 168
McGill University .......113, 515
Salvation Army Hospital .... 217
Tuberculosis Hospital (Bath) 217
Tulane Medical Department. 514

Congress of Buenos Aires ....... 399
Conway, M. P., filtration of polluted water supply ............ 423

**Correspondence:**

Buffalo Fresh Air Mission, milk committee—A. C. Goodyear .................. 264
Controlling nursing—Eugene Underhill ................. 209
Division of Fees—John H. Pryor ..................... 563
Dr. Jacobi's birthday—Franklin C. Gram ............... 505
Obstetrical teaching in Europe and America—B. C. Hirst ..................... 210
Paris and Germany—J. M. Mathews ............ ... 153
Preservation of medical records—Charles A. L. Reed. 152

Creveling, J. P., gunshot lesion of spinal column ................ 475
Crocker, George, gift to Columbia University ................ 327

DANFORTH, W. C., antigonococcic serum, report of cases 13
Deaver, John B., modern surgery of the stomach ......... 78
reception to ... 326
Dermatitis, epidemic of an urticaroid ........................ 96
Diabetes, treatment of .......... 99
Dowd, J. Henry, genitourinary specimens .................. 652
Dunnington, F. P., tincture of white soap ................... 197

EAT too much ................. 46
Education, menace to ...... 581

Page

FEES, dividing ................ 465
pertaining to ......... 384
Ferryboat for consumptives .108, 269
Flat foot, and skin lesions ....... 66
Flies, crusade against ........46, 106
Fort, Governor, condemns medical expert witness ............. 107
Fourth of July, a quiet .......... 570
Frost, Henry P., two cases of brain tumor ................ 5

GALL-BLADDER surgery ... 175
Gallstone disease .......... 253
treatment of ..... 297
Genitourinary specimens ........ 652
Gift, his ......................... 407
Gunshot lesion of spinal column. 475
Gynecological practice .......... 357

HARTEL, FRITZ, technic of venous anesthesia ......... 552
Hayd, Herman E., gall-bladder surgery .. 175
treatment of prolapsus uteri 291
Hay fever ........................ 663
Hays, Harold, studies of the nasopharynx ...................... 249
Headaches ...................... 230
Health, department of public .... 523
Heart, effect of steamer vibrations on ........................... 48
Hookworm disease gift ......... 270
Hookworm, report on .......... 509
Hopkins, Henry R., poise of scholarship ................... 1
Hornell tuberculosis hospital .... 510
Hydrophobia, death from ....... 571
new treatment for. 572

**Illustrations:**

Bull, solution of milk problem:
Figs. 1, 2, 3, 4, milch goats ............234, 236, 237, 238
Dowd, genitourinary specimens:
Fig. 1, tumor of bladder... 653
Fig. 2, wax taper ......... 653
Figs. 3 and 4, bladder stones 654
Fig. 5, lobes of prostate... 655
Hayd, treatment of prolapsus uteri:
Fig. 1, posterior vaginal wall .............. 294
Fig. 2, buried sutures .... 295
Fig. 3, wound sutures .... 295
Fig. 4, skin sutures ....... 296
Fig. 5, completed operation 297

Page

Hays, electric pharyngoscope 351
Howe, Lucien, decoration for 211
Krauss, William C., portrait.. 159
Meisenbach, talipes equino varus:
Figs. 1 and 2, congenital club-foot ............190, 191
Fig. 3, club-foot shoe ..... 193
Figs. 4 and 5, paralytic club-foot ..................194, 196
Wende, Ernest, portrait ..... 448

Insanity, tests of in courts....... 432

**Items:**

Antikamnia Chemical Co., Calendar, 1910 ............ 348
Apollinaris .................... 290
Battle & Co., charts 116, 174, ........................348, 522
Breitenbach, M. J. Co., Year Book, 1910 ............... 348
Calox ....................... 348
Denver Chemical Mfg. Co., vs. Colorado Chemical Co. 522
Farbenfabriken of Elberfeld Co., Aspirin patent ....... 174
Leighton-Wright Co. ........ 684
Moet and Chandon White Seal Champagne .......... 290
Schering & Glatz, removal .. 684
Tarrant's Seltzer Aperient... 348

JACOBI, ABRAHAM, sixty years of practice ........... 560
Jacobson, Nathan, infantile scurvy 362
Jonnesco, Thomas, stovaine ..... 326

KIDNEY, surgical ............ 76
King Edward, friend of medical profession ................. 627
King, James E., gynecological practice ................. 357

LAPARATOMY, abdominal supporters after ............ 187
Leaf, Cecil H., surgical treatment of cancer of rectum ........... 127
Leitner, George A., treatment of hay fever ...................... 663

**Leading Articles:**

American Association of Obstetricians and Gynecologists ...................... 212
Antivaccination crusade ..... 395
Appointments by Health Commissioner Fronczak ... 570
Bureau of Public Health.... 324
Budapest Congress .......... 44
Cheerful sick room .......... 506
Division of fees ............. 508
Edward VII ................ 623
Famous English physicians . 567
Howe, Lucien, decorated .... 211
Infectivity of tabardillo ..... 451
Krauss, William C. .......... 158
Lawyers and doctors ....... 396
Mann, Matthew D. .......... 667
Mayor Adam's gift to Buffalo 325
New health commissioner ... 508
North pole reached ......... 160
Pellagra, treatment of ....... 162
Prevention of gonorrheal infection ................... 266
Reception to Dr. Jacobi...... 625
Second International Conference on Leprosy .......... 268
State control of medical practice, limit of .............. 104
University of Buffalo Commencement ............... 670
Wende, Ernest .............. 447

Lederle, Ernst J., commissioner of health ..................... 511
Legal rights of doctors ......... 319
Leprosy, lecture on ............. 504

**Literary Notes:**

American Journal of Surgery 288
American Journal of Physiologic Therapeutics ........ 521
American Journal of Physiologic Therapeutics ........ 637
Annals of Surgery, July, 1909, 58; Dec., 1910 ............. 346
Cincinnati Lancet-Clinic .... 464
Genitourinary Facts—J. Henry Dowd ............. 637
Hudson-Fulton celebration .. 406
Medical Libraries ........... 346
Medical Review of Reviews.. 522
New England Medical Monthly ................. 637
Physicians' Business Journal. 522
Saunders, W. B. Co.—Illustrated catalogue .......... 57
Saunders, W. B. Co.—illustrated catalogue .......... 464
Trained Nurse ..........116, 174
Wilson's Medical Diagnosis—J. B. Lippincott Co. ......... 57

Lucid, M. M., post-operative tetanus ...................... 305

Page

MANN, MATTHEW D., dividing professional fees... 465
Mann, Matthew D., address to graduating class U. of B. ..... 639
McGuire, Francis W., treatment of peritonitis ................. 596
Medical expert witness condemned 107
records, preservation of . 163
expert ................. 257
Medinal in mental disease ....... 103
, hypnotic ............... 387
Meisenbach, R. O., talipes equino varus ......................... 189
Metropolitan Life Insurance Co.'s brochure .................... 397
Midwifery, examiners in, Erie County ....................... 214
Military Surgeon, July, 1909 ..... 163
Milk problem .................. 233

**Miscellany:**

Army Medical Corps examinations ..................289, 580
Medical supervisor .......... 232
Pharmacopeial Convention, 1910 ..................... 638
Public Health and Marine Hospital Service examinations ...................... 347
Radu Surgical Instrument Co. ........................580, 684
State Civil Service examinations ..................... 347
Squirrels as plague carriers.. 58
U. S. Civil Service examinations ..............579, 637, 638

Morris, Robert T., abdominal supporters after laparatomy ....... 187

NASOPHARYNX, studies of.. 349
Nervous sequelae of infectious diseases ................. 68
Norton, Roy, his gift ............ 407

OBSTETRICS, teaching of 614, 656

**Obituary:**

Atkinson, William B. ....... 329
Bailey, Joseph G. ........... 629
Beach, Eugene ............... 50
Blackman, D. E. ............ 272
Blackwell, Elizabeth ........ 678
Bowden, James Wylie ....... 629
Congdon, Lemier ........... 272
Crans, A. F. ................ 216
Crittenton, Charles N., Esq.. 274
Dambach, John ............. 165
Denton, Lewis A. ........... 629
Dewey, Frank E. ............ 329
Dolley, Sarah R. A. ......... 454
Elsner, Simon L. ........... 678
Foote, John ................ 110
Griffin, William T. .......... 110
Gumaer, Adelbert G. ........ 166
Hagey, Jacob M. ........... 110
Hamilton, David ............ 215
Highland, Lawrence A. ..... 572
Howe, Carey W. ............ 50
Knapp, James C. ............ 328
Koch, Robert ............... 677
Krauss, William C. ......... 158
Lea, Henry Charles, Esq. .... 272
Matteson, George Washington ...................... 572
McNett, George C. .......... 272
Palmer, Elmore ............. 272
Parkhill, Clair S. ............ 110
Pierce, Alfred P. ............ 50
Pierce, J. H. ................ 571
Piffard, Henry Granger ..... 677
Pitt, William H. ............ 109
Seymour, George ........... 271
Smith, Andrew H. .......... 573
Spencer, Thomas D. ........ 572
Spencer, James D. .......... 629
Swift, Ora Clay ............ 512
Wende, Ernest ............. 447

Owen, R. L., department of public health ...................... 523
Oysters, not "floated" ...389, 452, 571

PANCREAS, cancer of ........ 157
Partnerships among physicians ...................... 321
Patella, fracture of ............. 248
Pellagra .....................164, 198
Peritonitis, treatment of ........ 596
President, undignified reference to 675

**Personal:**

Abrams, Edward T. ......... 108
Bennett, A. G.; Bingham, H. H.; Dorr, L. Bradley; Smith, Chauncey Pelton; MacDonald, Carlos F. ..... 399
Breuer, Max; Sheehan, Robert F.; Sy, Albert P.; Carroll, Jane W.; Keyes, Regina Flood; Fronczak, Francis E.; Wende, Ernest; Gregory, Willis G.; Mansperger, William ................. 49
Briggs, A. H.; Renner, W. S.; Knisley, A. B.; Gardner, James A.; Long, Charles E.; Bemus, Morris N.; Dorr, L. Bradley .......... 271

Page

Fairbairn, J. F.; Gardner, James A.; Schroeter, L.; Haskell, Clayton K.; Jewett, Carlton R.; Briggs, A. H. .. 627
Glosser, H. H.; Russell, Nelson G.; Hengerer, A. W.; Wiley, Harvey W.; Roberts, Carroll J.; Park, William H.; Getman, William T.; McCormack, J. N. ........ 628
Gunn, Lee; Bagley, Thomas; Gilray, E. J.; Baketel, H. Sheridan .................. 511
Heyd, Charles Gordon ...... 629
Howard, Charles F.; Calhoun, J. C.; Wilgus, Sidney J. ... 572
Jacobi, A. .................. 453
Lewis, F. Park; Frost, Henry P. ....................... 512
McKenney, D. C.; Murphy, John B.; Hodge, William H.; Carmichael, A. D. ..... 454
Miller, A. B.; Glosser, H. H.. 164
Murphy, John B.; Schwarz, Henry; Crile, George W.; Vander Veer, Edgar A. ... 677
Noehren, Alfred H.; Grant, John H.; Himmelsbach, George H. ................ 109
Pryor, John H. ............. 47
Rafter, John A. ............. 214
Rice, James F.; Chase, Walter B.; Frye, Maud J., Marcy, William H.; Tinkler, John; Shanahan, William T.; Hyde, Clarence L., Grove, W. G. ............. 215
Robinson, Ralph; Hopkins, Henry R.; Putnam, James W. ...................... 48
Sloan, George A.; Wilson, Nelson W.; Garvin, Albert H.; Carr, Joseph H.; Palmer, Charles N. ........... 328
Walker, H. D.; Pease, Herbert D.; Burkhardt, F. W.; Williams, H. U.; McMurtry, L. S.; Lobenstine, Ralph Waldo ............. 165
Wright, Adam H.; Potter, Julius H. ................. 676

Phipps, Henry, tuberculosis gift.. 327
Physicians' office building ....... 452
Placenta previa, mechanism and treatment of . 15
treatment of ... 69
Prolapsus uteri ................. 291
Putnam, James Wright, nervous sequelae of infectious diseases.. 68

**Progress in Medical Science:**

Medico-legal, by John A. Rafter .......257, 319, 384, 432

Page

QUACKS, why prosper ....... 510

RAFTER, JOHN A., medical man of China ............. 477
Rankin, Guthrie, headaches and their treatment ............... 239
Reed, Charles A. L., referee board on benzoate of soda ........... 117
Red Cross stamps ............... 511
Regents' examinations, new law.. 625
Reich, A., veronal in vomiting of pregnancy .................... 438
Roosevelt Hospital ............. 214
Ross, James F. W., fibromyomatous tumors ................... 601

SANATORIUM, Queen Alexandra ...................213, 217
Sarcoma, melanotic ............. 430
Scholarship, poise of ............ 1
Schools and school buildings, report of commission on... 95
for tuberculosis children 210
Schwarz, Henry, mechanism and treatment of placenta previa 15
treatment of placenta previa 69
Scurvy, infantile ................ 362
Serum, antigonococcic .......... 13
Sick and injured, care of ........ 182

**Society Meetings:**

American Association of Obstetricians and Gynecologists ...................110, 166
American Academy of Medicine ..................273, 630
American Medical Association ........................574, 678
American Gastroenterological Association ............ 630
American Hospital Association ..................... 678
American Proctologic Society 574
Association of American Medical Colleges .......... 459
Buffalo Academy of Medicine 50, 220, 273, 333, 400, 459, ..................512, 573, 630
Council on Medical Education 331
Dental Societies ............. 218
Groszmann School .......... 513
Homeopathic Medical Society, Western N. Y. ........... 573
International American Congress of Medicine and Hygiene ..................... 400
Lake Keuka Medical and Surgical Association ...... 112

Page
Medical Society State of New York ..................... 328
Medical Society State of New York, Eighth District Branch ..................... 163
Medical Association of Central New York ........... 218
Medical Society County of Chautauqua ............... 332
Medical Society County of Erie ..........220, 331, 573, 679
Medical Society County of Wayne .................... 332
Mississippi Valley Medical Association ............... 167
National Confederation State Medical Examining and Licensing Boards .....514, 630
Psychological Congress ..... 113
Rochester Academy of Medicine 273, 333, 401, 458, 513, ........................574, 631
Southern Surgical and Gynecological Association ..219, 401
United States Pharmacopeial Convention ................ 454
University Convocation, State of New York ............. 166

**Society Proceedings:**

American Proctologic Society ........................ 82, 135
Buffalo Academy of Medicine 38
Chicago Medical Society..... 383
Medical Society County of Erie ......30, 254, 369, 482, 610
National Confederation State Medical Examining and Licensing Boards ......... 34

Soap, tincture of white .......... 197
Spitting on sidewalks, dangers of 646
Spofford, H. M., chronic cholecystitis ....................... 10
Stevenson, F. H., nervous and mental manifestations due to arteriosclerosis .... 416
Stomach, surgery of ........... 78
Sugar for fatigue ............... 107

TALIPES equino varus ....... 189
Tetanus, post-operative .... 305
Thermos bottle, infection from.. 453
Tinea versicolor, cure of ......... 43
Tinker, Martin B., surgical treatment of gallstones ............ 297

Page
**Topics of Public Interest:**

Appointments by regents.... 665
Bureau of census ........... 499
Common colds .............. 498
Consumptives, four thousand starve yearly ............. 148
Guiteras, Ramon, South Africa trip .................... 150
Metropolitan Life Insurance Company's hospital ....... 443
Mortality statistics of next year ...................... 206
Municipal Hospital ......... 390
Red Cross stamps .......... 322
Schools in open air .....618, 666
State board of pharmacy..... 666
Tuberculosis, appropriations to prevent ............... 149
Tuberculosis conference .... 501
Tuberculosis crusade ....393, 621
Tuberculosis law ........... 444
Tuberculosis preventorium for children ............... 261
Tuberculosis Sunday ........ 445

Toxins and the liver ............ 590
Treves, Sir Frederick, upholds vivisection .................. 47
Tuberculosis commission, report of ................ 38
children, schools for 210
ferryboat for cases.. 108
lecture on ......... 397
Tumors, fibromyomatous ........ 601
"Typhoid Mary," isolated ....... 45

VAN PEYMA, P. W., present menace to education .. 581
report of commission on schools and buildings ....... 95
Veronal in vomiting ............ 438
Vesical inflammation in female .. 42

WALLACE, W. L., melanotic sarcoma ................. 430
Water supply, filtration of ....... 423
Wiley, Harvey W., receives decoration ........................ 107
Wireless, prescription by ....... 47

YOUNG, J. C., care of sick and injured ..................... 182

